# Integrative Multi−Omics Analysis Reveals Candidate Biomarkers for Oral Squamous Cell Carcinoma

**DOI:** 10.3389/fonc.2021.794146

**Published:** 2022-01-14

**Authors:** Zhengqing Wan, Haofeng Xiong, Xian Tan, Tong Su, Kun Xia, Danling Wang

**Affiliations:** ^1^ Hengyang Medical School, University of South China, Hengyang, China; ^2^ The Affiliated Changsha Central Hospital, Hengyang Medical School, University of South China, Changsha, China; ^3^ Postdoctoral Station for Basic Medicine, Hengyang Medical School, University of South China, Hengyang, China; ^4^ Xiangya Hospital, Central South University, Changsha, China; ^5^ Center for Medical Genetics, School of Life Sciences, Central South University, Changsha, China

**Keywords:** oral squamous cell carcinoma, multi-omics, DEGs, DMGs, MeDEGs

## Abstract

Oral squamous cell carcinoma (OSCC) is one of the most common types of cancer worldwide. Due to the lack of early detection and treatment, the survival rate of OSCC remains poor and the incidence of OSCC has not decreased during the past decades. To explore potential biomarkers and therapeutic targets for OSCC, we analyzed differentially expressed genes (DEGs) associated with OSCC using RNA sequencing technology. Methylation−regulated and differentially expressed genes (MeDEGs) of OSCC were further identified *via* an integrative approach by examining publicly available methylomic datasets together with our transcriptomic data. Protein−protein interaction (PPI) networks of MeDEGs were constructed and highly connected hub MeDEGs were identified from these PPI networks. Subsequently, expression and survival analyses of hub genes were performed using The Cancer Genome Atlas (TCGA) database and the Gene Expression Profiling Interactive Analysis (GEPIA) online tool. A total of 56 upregulated MeDEGs and 170 downregulated MeDEGs were identified in OSCC. Eleven hub genes with high degree of connectivity were picked out from the PPI networks constructed by those MeDEGs. Among them, the expression level of four hub genes (CTLA4, CDSN, ACTN2, and MYH11) were found to be significantly changed in the head and neck squamous carcinoma (HNSC) patients. Three hypomethylated hub genes (CTLA4, GPR29, and TNFSF11) and one hypermethylated hub gene (ISL1) were found to be significantly associated with overall survival (OS) of HNSC patients. Therefore, these hub genes may serve as potential DNA methylation biomarkers and therapeutic targets of OSCC.

## Introduction

Oral squamous cell carcinoma (OSCC) is the seventh most common cancer in males under the age of 60, with about 400,000 new cases reported annually worldwide (1). Geographically, the incidences of OSCC in developing countries like India and Brazil could be more than tenfold higher than those in Western countries. Although OSCC is predominantly seen in male over the age of 65, the disease appears to be increasing in younger patients under the age of 45 and the gender difference has been decreasing over the past few decades. Thus, OSCC represents a major public health problem, especially in poor and developing countries.

Despite recent advances in therapeutic modalities, the five−year morbidity rate of OSCC remains high and the survival rate stays dismal. Under the standard treatment plan, the outcomes of patients with OSCC could be completely different (2, 3). Therefore, uncovering the complex molecular changes leading to the development of OSCC as well as the factors contributing to the differential prognosis among OSCC patients represents a continuingly unmet medical need.

The etiology of OSCC is complicated and involves both genetic and environmental factors (4). DNA methylation is one of the most abundant epigenetic modifications with key regulatory functions in gene transcription and genomic stability. Growing evidence suggests that alterations of DNA methylation contribute to carcinogenesis. In OSCC, both global and site−specific DNA hypomethylation have been reported (5–7). Kim et al. found that three out of 14 tumor suppressor genes (TSGs) are hypermethylated and transcriptionally silenced in OSCC (8). Using microarray−based technologies, the methylomic alternations in OSCC have been extensively studied and reported by multiple groups (5, 9–15). Considering the fact that the majority of genes that are abnormally methylated in cancers have no corresponding changes in mRNA expression, it becomes important to understand the functional profile of these massive genome−wide methylomic data. Two recent studies have taken a meta−analysis approach and analyzed the impact of DNA methylation on gene expression and biological pathways using publicly available transcriptome and methylome datasets (16, 17). Due to the unknown heterogeneity within and across individual studies with regard to clinical diagnosis, tumor tissue dissection, sample processing, etc., more research efforts are needed to gain a trustworthy assessment of methylomic signatures in OSCC.

In this study, we have characterized the transcriptome of fresh OSCC tumor and paired normal adjacent tissue (NAT) using RNA sequencing technology. Two methylation−profiling datasets, GSE38532 and GSE46802, generated from the same microarray platform and of paired case−control design, were selected and integrated with our transcriptome data for multi−omics analysis. Methylation−regulated and differentially expressed genes (MeDEGs) were identified, hub genes were screened through protein−protein interaction (PPI) network analysis, and further validated in The Cancer Genome Atlas (TCGA) database. Using this integrated approach, we were able to identify novel DNA methylation regulated genes with potential clinical applications in OSCC.

## Materials And Methods

### Tumor Biospecimen Collection and RNA Extraction

In total, 17 paired samples of OSCC and NAT were collected from newly diagnosed patients who underwent surgical resection and had received no prior treatment for the disease at Xiangya Hospital of Central South University (CSU). The samples were collected in accordance with the guidelines issued by the Ethics Committee of School of Life Sciences, CSU (Record#2020−1−42). Written informed consent was obtained to all participants in this study.

All biospecimens from patients were pathologically reviewed and confirmed by two independent pathologists. Among these patients, there were four with stage I, four with stage II, four with stage III, and five with stage IV. Their demographic characteristics are presented in **Table 1**. After surgical removal, all samples were immediately transferred to the lab on ice using CO_2_ independent medium (18045088, Gibco, USA). Tissue blocks of about 30 mg were further dissected out and used for extraction of total RNA following the manufacturer’s instruction of the AllPrep DNA/RNA/miRNA Universal Kit (80224, Qiagen, USA).

**Table 1:** Patient demographics and clinical characteristics.

Characteristics	Parameters	No. of patients
Age (years)	<45	4
	46~60	8
	>61	5
Gender	Male	16
	Female	1
Stage	I	4
	II	4
	III	4
	IV	5
Degree of differentiation	Well differentiated	8
	Moderately differentiated	7
	Poorly differentiated	1
	ND^a^	1
Cancer site	Tongue	8
	Gingiva	3
	Inner cheek	6
P53 expression	Positive	5
	Negative	5
	ND	7
Ki67 expression	>60% positive	3
	30%~60% positive	5
	<30% positive	3
	ND	6

a ND, Not determined.

### RNA Sequencing and Data Processing

For library preparation, 1 μg of total RNA from each sample was depleted for rRNA using the Ribo−Zero rRNA Removal Kit (RZH1046, EPICENTRE, USA), followed by construction of sequencing library using the TruSeq RNA Sample Prep Kit v2 (RS−122, Illumina, USA). Individual RNA libraries were pooled and sequenced using paired−end sequencing with 150 bp reads on an Illumina HiSeq3000 instrument.

Adapter and low−quality reads were first trimmed using Trim Galore (version 0.4.4) in paired−end mode with default parameters. Trimmed reads were then mapped to the hg19 reference genome to count mapped reads for all human transcripts. Normalization and analysis of differentially expressed genes (DEGs) were carried out using multifactor analysis (tumor vs. NAT) in the DESeq2 R program package (version 1.30.1). Significant genes were identified with statistical cut−off of |Log_2_FC| > 2 and false discovery rate (FDR) < 0.05.

### Methylation Data Acquisition and MeDEGs Identification

Microarray−based gene methylation profiling datasets GSE38532 and GSE46802 were obtained from the NCBI gene expression omnibus (GEO, https://www.ncbi.nlm.nih.gov/geo/). Forty OSCC and 40 matched NAT specimens were obtained in GSE38532, while ten paired OSCC and NAT specimens were included in GSE46802. Both microarrays used the Illumina GPL8490 microarray platform (HumanMethylation 27 BeadChip, HumanMethylation27_270596_v.1.2, Illumina, USA). This methylation chip contains 27,578 individual CpG sites spread across the proximal promoter regions, defined as 1500 bp upstream of the transcription start site (TSS1500), of 14,495 genes.

The GEO2R online software was utilized to analyze these two datasets to identify differentially methylated genes (DMGs). FDR < 0.05 and delta *β*−value > 0.1 were used as the cut−off standards during analysis as previously described (18). Eventually, Venndiagram package (version 1.6.20) of R program was used to obtain the overlaps between DMGs and DEGs and to identify MeDEGs, including downregulated genes with a hypermethylated pattern and upregulated genes with a hypomethylated pattern.

### Functional Enrichment Analysis

Functional clustering annotation of the DEGs and MeDEGs were conducted *via* the Metascape online tool (19). For functional clustering, enriched annotations from the Gene Ontology (GO) biological processes and the Reactome gene sets were included. Analyses were carried out with the default parameters of minimal overlap of 3, minimal enrichment ratio of 1.5, and *p*−value < 0.01 as previously described (20).

### Protein−Protein Interaction (PPI) Network Construction

The PPI networks of downregulated genes with a hypermethylated pattern and upregulated genes with a hypomethylated pattern were constructed using the Search Tool for the Retrieval of Interacting Genes (STRING, version 11.0) online database. PPI with an interaction score > 0.4 was visualized in the final STRING network. Protein networks are clustered to MCL inflation parameter of 2 (21).

### Identification of Potential Hub Genes

To identify potential hub genes, the cytoHubba plugin of the Cytoscape (version 3.8.2, http://www.cytoscape.org/) software was used to screen modules within PPI network. Four topological analysis methods were individually utilized (MCC, MNC, Degree, and EPC) to obtain the top ten important genes from each PPI network. Common genes selected from all four methods were regarded as potential hub genes with consensus.

### Validation of Hub Genes

The Gene Expression Profiling Interactive Analysis (GEPIA) online platform provides fast evaluation between the survival effect and the expression profile analysis of DEGs in a given cancer type. To validate aforementioned hub genes, the relative expression levels of these genes in head and neck squamous cell carcinoma (HNSC) were identified with statistical cut−off of |log_2_FC| > 1 and *p*−value < 0.05 as previously described (22). In addition, the overall survival (OS) effect of hub genes in HNSC was estimated by calculating the log−rank *p*−value and the hazard ratio (HR). Patients were divided into high (higher than the 75^th^ percentile) and low (lower than the 25^th^ percentile) expression groups based on the normalized expression level of the selected gene across all HNSC patients in the database (23). Since there is no OSCC category in both GEPIA and TCGA databases, and OSCC accounts for ~95% of HNSC, we chose HNSC as the category for these validation analyses.

### Immune Infiltration Analysis

Infiltrations of immune cells in OSCC tumors and NATs were estimated using TIMER web tool (24, 25). Briefly, RNA−seq data in TPM (transcripts per million) format from the 17 paired samples of OSCC and NAT were calculated. The infiltration abundance of B cell, CD4^+^ T cell, CD8^+^ T cell, neutrophil, macrophage, and myeloid dendritic cell in each sample were evaluated using TIMER2.0. Similar procedures were used to analyze data from TCGA-HNSC and paired NAT samples.

## Results

### Characterization of RNA Sequencing and Mapping

Using RNA sequencing, a total of 1418 million and 1289 million cleaned reads were obtained from pooled OSCC and NAT libraries with 65−106 million reads per library for all samples. In all 34 libraries, > 92.9% cleaned bases have Phred quality score (Q−scores) above 30, indicating a high base call accuracy. Forty-two to eighty-one million reads were uniquely mapped to the hg19 reference genome for each sample, with an average unique mapping ratio of 92% (**Table 2**).

**Table 2:** RNA sequencing profile.

	Sample	Clean reads	Q30 rate^a^	Clean reads (no rRNA)	Unique mapped^b^	Unique mapping ratio
Oral cancer samples	C1	86871496	93.80%	73156876	66641472	93%
	C2	95042470	94.45%	91337822	81376448	92%
	C3	77878418	93.85%	64697966	48192246	81%
	C4	94390710	94.50%	89400168	77485932	93%
	C5	86429488	94.05%	74299012	67319063	93%
	C6	84077700	93.80%	77808674	60775290	94%
	C7	89995568	93.85%	82408242	73256107	92%
	C8	76815890	94.10%	74169890	51440016	83%
	C9	69672812	93.50%	60281976	51178965	88%
	C10	73618490	93.00%	69337410	57362728	96%
	C11	73679338	93.90%	70965340	62125998	91%
	C12	69570852	94.05%	67902214	61896005	93%
	C13	71761106	93.60%	63710848	54846328	90%
	C14	86873658	94.25%	81753826	73884842	93%
	C15	106027960	94.45%	91483292	81696922	91%
	C16	91772898	94.40%	83796796	70456741	92%
	C17	83459218	93.65%	79658790	70201492	90%
Matched adjacent normal tissues	N1	69157408	93.50%	67681854	63645851	96%
	N2	65305948	94.00%	63998054	51351866	92%
	N3	67139172	93.70%	64235992	47105221	90%
	N4	76287040	93.65%	65877516	53256262	93%
	N5	78444622	93.60%	72012974	64120834	92%
	N6	71185918	93.85%	63433540	56272465	94%
	N7	64770514	93.85%	63831196	52469386	97%
	N8	83902570	94.25%	82497916	75099818	95%
	N9	71547434	93.95%	69248746	63107749	94%
	N10	77368874	92.90%	67161816	59603271	94%
	N11	87521000	93.70%	81245060	72857823	94%
	N12	65356658	93.65%	62147174	54454670	94%
	N13	90320998	94.15%	88577280	58883887	94%
	N14	100126302	93.85%	90108998	80048283	94%
	N15	67877166	94.15%	66695120	60492545	95%
	N16	70708242	93.60%	67644350	42576164	90%
	N17	81724362	93.50%	77076054	71227418	95%

a Q30 rate: The percentage of base with a Phred value > 30.

b Unique mapped: The number of clean reads that mapped onto the hg19 reference genome.

### Identification of DEGs in OSCC

**Figure 1:**
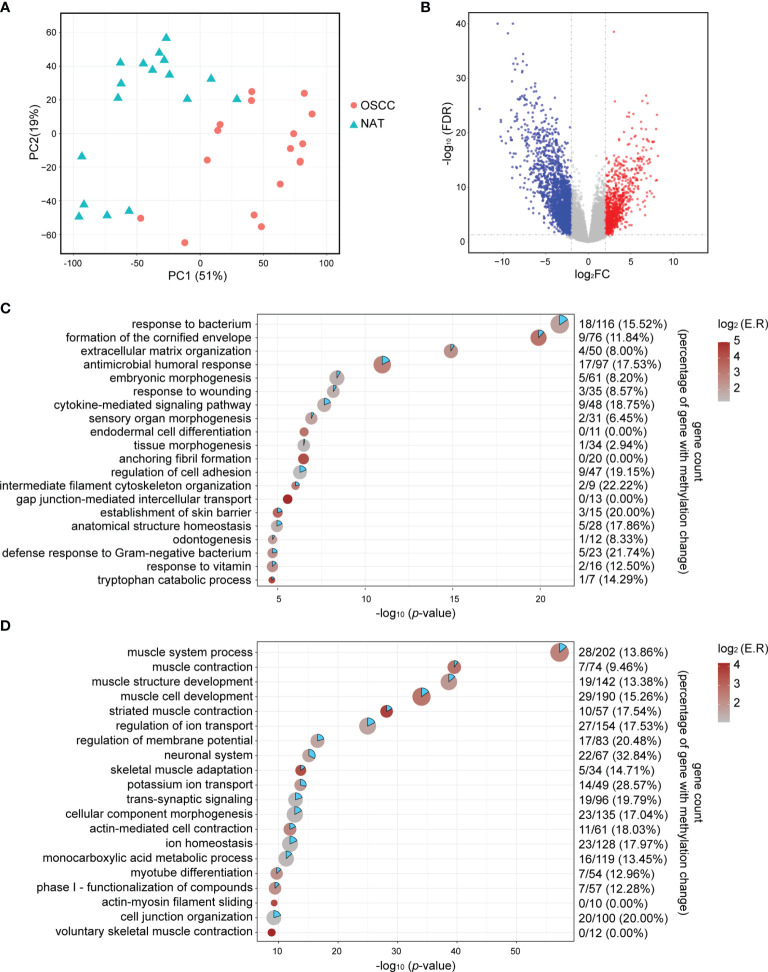
Identification of DEGs. **(A)** PCA plots of RNA-seq data show the different characteristic of RNA expression patterns of OSCC tissues and NATs. Each dot indicates a sample. **(B)** Volcano plot visualizing the DEGs identified by the DESeq2 packages. The vertical dotted lines are positioned at log_2_FC of 2 or -2. The horizontal dotted lines are positioned at FDR = 0.05. **(C)** Point plot for the top 20 enriched functional clusters for upregulated genes. X-axis indicates -log_10_ (*p*-value), color indicates enrichment ratio, and size indicates gene counts in unique functional clusters. Gene count in the right panel indicates hypomethylated-upregulated genes/all upregulated genes (percentage of upregulated gene with methylation change). **(D)** Point plot for the top 20 enriched functional clusters for downregulated genes. X-axis indicates -log_10_ (*p*-value), color indicates enrichment ratio, and size indicate gene counts in unique functional clusters. Gene count in the right panel indicates hypomethylated-upregulated genes/all upregulated genes (percentage of upregulated gene with methylation change). log_2_FC, log_2_ transformed fold change; NAT, normal adjacent tissue; FDR, Benjamini & Hochberg adjusted *p*-value.

Principal component analysis (PCA) of RNA−seq data showed that deconvoluted data from OSCC samples and NATs were clustered into different patterns (**Figure 1A**), suggesting the underlying differences between these two conditions. A total of 778 upregulated genes and 2211 downregulated genes were obtained by the DESeq2 package using the cut−off criteria of FDR < 0.05 and |log_2_FC| > 2, as shown in **Figure 1B** by a volcano plot. Further functional clustering analysis using the online tools in Metascape shown that upregulated genes were mainly involved in functions such as response to bacterium, formation of the cornified envelope, extracellular matrix organization, antimicrobial humoral response, etc. (**Figure 1C**), while downregulated genes were mainly involved in muscle system process, muscle contraction, muscle structure development, muscle cell development, etc. (**Figure 1D**).

### Identification of Aberrantly MeDEGs in OSCC

**Figure 2:**
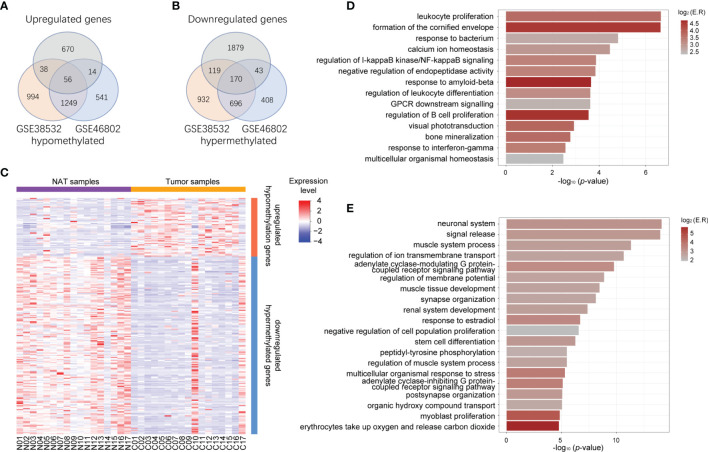
Identification of aberrantly methylated and differentially expressed genes. **(A)** Venn diagram of upregulated genes (grey circle) and hypomethylated genes common to both methylation datasets (orange circle for dataset GSE38532 and blue circle for dataset GSE46802). **(B)** Venn diagram of downregulated genes (grey circle) and hypermethylated genes common to both methylation datasets (orange circle for dataset GSE38532 and blue circle for dataset GSE46802). **(C)** Heatmap representing expression level of all the NAT and tumor samples (columns) and genes (rows) detected as methylation-regulated differentially expressed. **(D)** Bar plot represents functional clusters of upregulated-hypomethylated genes with statistical significance. X-axis indicates -log_10_ (*p*-value), color indicates log_2_ (enrichment ratio). **(E)** Bar plot represents functional clusters of downregulated-hypermethylated genes with statistical significance. X-axis indicates -log_10_ (*p*-value), color indicates log_2_ (enrichment ratio).

To identify potential MeDEGs (i.e., genes that exhibit inverse correlation between their alterations of DNA methylation at promoter regions and gene expression), we performed a multi−omics analysis by integrating our transcriptomic data and publicly available methylomic data (GSE38532 and GSE46802). As shown in the Venn diagrams in **Figures 2A, B**, 56 upregulated and hypomethylated genes and 170 downregulated and hypermethylated genes (**Table 3**) were identified from this study. The heatmap of these potential MeDEGs is demonstrated in **Figure 2C**.

**Table 3:** Methylation−regulated differentially expressed genes.

Upregulated and hypomethylated genes				Downregulated and hypermethylated genes					
ACY3	CST1	KRT6C	SERPINA10	ABCA3	CNKSR2	GLRB	LPL	PENK	SSTR1
AIM2	CTLA4	KYNU	SERPINB7	ABCC8	CNNM1	GPR26	LRFN5	PEX5L	ST6GAL2
ALOX12B	CYP27B1	LRRC15	SIGLEC12	ACADL	COL14A1	GPR27	LRRTM1	PHYHIPL	ST8SIA5
APOC2	DEFB103A	MFAP2	SLC44A5	ACSL6	COX7A1	GPR88	MEGF10	PODN	STAC2
ARL14	FCRL3	MMP13	SLC5A12	ACTA1	CRHR2	GRIA4	MLPH	POU3F3	SYN2
ATP6V0D2	GAST	MSLN	SLC6A7	ACTN2	CTTNBP2	GRIK3	MT1A	PPP1R9A	TACR1
C11orf44	GPR29	PI3	SPOCD1	ADCY5	CYP26C1	GRM6	MYH11	PTGFR	TCF15
CASP14	GPR39	PLA2G2D	TM4SF19	ADCYAP1R1	DES	HAND2	MYO3A	PTGIS	TDRD5
CCR8	GRM4	PLA2G2F	TNFRSF13B	ADHFE1	DIO3	HBA1	MYOCD	PTPRT	THBS4
CD80	GRM5	PPEF1	TNFSF11	ADRA1A	DLK1	HBA2	MYOD1	RAB3C	TMEFF2
CDSN	HIST1H2BO	PSORS1C2	TNIP3	ADRA2A	DOK5	HCN4	MYRIP	RASL12	TNS1
CHRNA9	IL24	PTHLH	TREM2	ADRA2B	EDN3	HHIP	NCAM2	RBP4	TRPM3
CNGB1	KRT16	PTPRH	UBD	AGTR1	EEF1A2	HPSE2	NDRG2	RELN	TSPYL5
CPA6	KRT6B	S100A12	WFDC12	ALDH1A2	ELN	HS3ST2	NELL1	RGN	VGLL2
				ALDH5A1	EPDR1	HTR4	NOVA1	RIC3	VIPR2
				ARHGDIG	EPHA5	ID4	NT5C1A	RIMS4	WDR17
				BMP3	EPHA7	IGF1	NTRK3	RORC	WIF1
				CA4	EYA2	ISL1	OSR1	SCG3	WNK2
				CA8	EYA4	KCNA1	PABPC5	SFRP1	ZBTB16
				CAB39L	FAM3B	KCNA2	PALM	SHD	ZFP28
				CACNG6	FGF10	KCNA5	PAX7	SIM1	ZNF135
				CCDC60	FOXD3	KCNB1	PCDH11X	SLC16A12	ZNF471
				CDO1	GABBR2	KCNIP1	PCDH11Y	SLC27A6	ZNF660
				CFTR	GABRA2	KCNK12	PCDHA13	SLC6A4	ZNF667
				CGNL1	GALR1	KCNN2	PCDHAC1	SLITRK3	ZNF677
				CHRDL2	GCM2	KCTD8	PCDHAC2	SLITRK4	
				CKMT2	GDF10	KIF1A	PDE4DIP	SLITRK5	
				CLDN11	GFRA1	LDOC1	PDGFD	SORCS1	
				CLIC6	GFRA3	LMX1A	PEG3	SOX17	

### Functional Enrichment Analyses of Potential MeDEGs

To evaluate the biological functions of these potential MeDEGs, functional clustering was analyzed using the online tools in Metascape. As shown in **Figures 2D, E**, genes that were upregulated and hypomethylated were enriched in biological processes such as leukocyte proliferation, formation of the cornified envelope, response to bacterium, calcium ion homeostasis, etc., while downregulated and hypermethylated genes were linked to neuronal system, signal release, muscle system process, regulation of ion transmembrane transport, etc. To further understand the contribution of DNA methylation to the altered gene expression in OSCC, we evaluated the ratio of DEGs that were inversely affected by methylation changes in each enriched functional cluster. As shown in **Figures 1C, D**, differentially upregulated genes involved in functions such as intermediate filament cytoskeleton organization, defense response to Gram−negative bacterium, establishment of skin barrier, and regulation of cell adhesion were often hypomethylated (22.22%, 21.74%, 20.00%, and 19.15%, respectively). For differentially downregulated genes, those involved in neuronal system, potassium ion transport, regulation of membrane potential, and cell junction organization were most likely to be hypermethylated (32.84%, 28.57%, 20.48%, and 20.00%, respectively).

### PPI Network Construction and Hub Gene Identification

**Figure 3:**
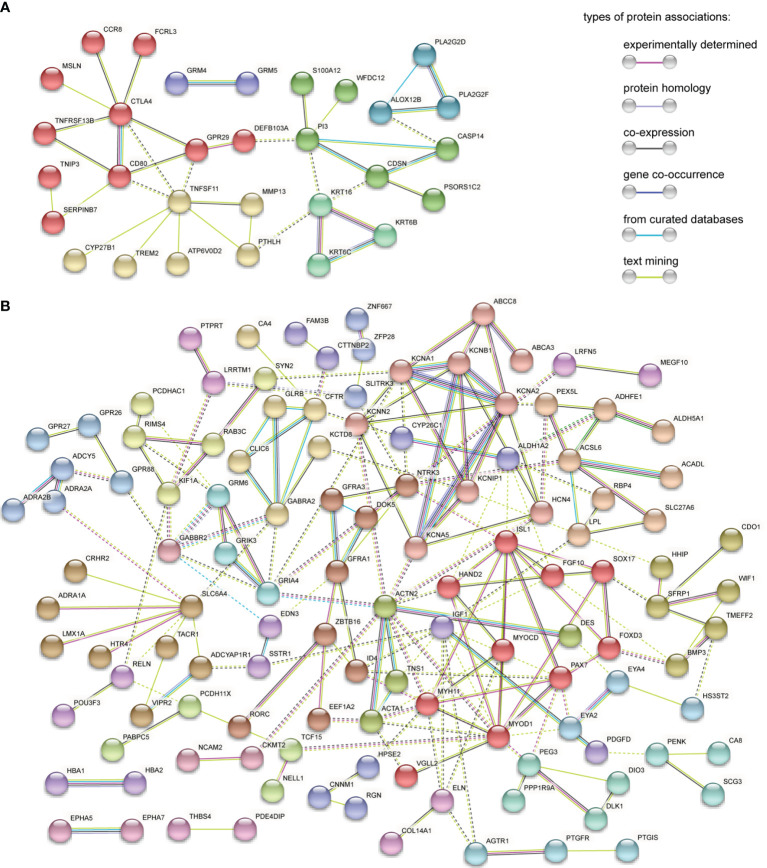
PPI network analysis of MeDEGs. **(A)** PPI network of upregulated and hypomethylated genes. **(B)** PPI network of downregulated and hypermethylated genes. Each node represents a protein coded by unique genes, edges indicate interactions, and the color of edge indicates the degree of associations between proteins. Disconnected nodes in the network were hidden. Protein networks are clustered on MCL inflation parameter 2. Nodes from the same cluster are presented in the same color. Edges between different clusters are represented by dashed line.

**Figure 4:**
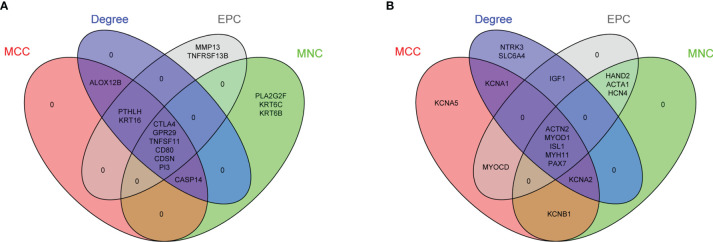
Identification of potential hub genes from PPI networks of MeDEGs. **(A)** Venn diagram of highly connected genes identified by four calculation methods from PPI network of upregulated and hypomethylated genes. Six overlapping genes were identified in the intersection of all lists. **(B)** Venn diagram of highly connected genes identified by four calculation methods from PPI network of downregulated and hypermethylated genes. Five overlapping genes were identified in the intersection of all lists. List of calculation methods: MCC (red), MNC (green), Degree (blue), and EPC (gray).

The PPI network of 56 upregulated MeDEGs and 170 downregulated MeDEGs were constructed using the STRING v11.0 database and visualized *via* the Cytoscape software. Thirty nodes and 38 edges were identified from the network of upregulated MeDEGs (**Figure 3A**), and 121 nodes and 207 edges were found from the network of downregulated MeDEGs (**Figure 3B**). Within each PPI network, the top 10 highly interacted genes were evaluated using four different calculation methods (MCC, MNC, Degree, and EPC) individually. Common genes coming out of all four methods were selected as potential hub genes. Eleven hub genes, including six hypomethylated genes (CTLA4, GRP29, TNFSF11, CD80, CDSN, and PI3) and five hypermethylated genes (ACTN2, MYOD1, ISL1, MYH11, and PAX7) were identified from these networks (**Table 4** and **Figures 4A, B**).

**Table 4:** Expression and Methylation Profiles of Eleven Potential Hub MeDEGs.

Gene symbol	Description	Expression profile			Methylation probe ID	Distance to TSS^c^ (nt)	Methylation profile			
		log_2_FC^a^	FDR^b^	*p*–value			GSE38532		GSE46802	
							FDR	Delta*β*–value	FDR	Delta*β*-value
CTLA4	Cytotoxic T-lymphocyte associated protein 4	2.23	6.21E-06	4.83E-07	cg08460026	37	6.47E-05	-0.13	7.84E-05	-0.31
GPR29	C-C motif chemokine receptor 6	2.55	2.43E-03	4.92E-04	cg05824215	211	4.37E-15	-0.22	3.44E-04	-0.19
					cg13615963	264	/	/	2.35E-07	-0.19
TNFSF11	TNF superfamily member 11	3.28	3.28E-05	3.19E-06	cg21094154	326	2.58E-15	-0.22	1.53E-02	-0.18
					cg24222324	381	1.51E-07	-0.13	9.22E-04	-0.14
CD80	CD80 molecule	2.54	5.13E-06	3.89E-07	cg06509940	465	1.37E-04	-0.17	6.34E-04	-0.18
					cg21572897	628	1.56E-31	-0.32	8.95E-06	-0.25
CDSN	Corneodesmosin	7.20	8.62E-16	7.87E-18	cg08424423	35	5.07E-12	-0.16	6.61E-05	-0.24
					cg24735489	129	/	/	1.82E-02	-0.12
PI3	Peptidase inhibitor 3	4.28	8.81E-14	1.23E-15	cg09462575	13	5.81E-12	-0.18	/	/
					cg02442161	139	3.01E-19	-0.28	1.79E-07	-0.42
ACTN2	Actinin alpha 2	-8.15	8.45E-27	8.35E-30	cg16853982	410	2.61E-05	0.14	/	/
					cg21376883	433	4.50E-27	0.27	2.67E-02	0.17
MYOD1	Myogenic differentiation 1	-6.09	9.18E-13	1.49E-14	cg16519321	84	1.75E-10	0.16	1.04E-02	0.18
					cg07271264	182	3.76E-13	0.25	9.82E-03	0.23
					cg18555440	528	1.50E-32	0.32	8.79E-05	0.27
					cg24322623	729	1.14E-08	0.14	/	/
ISL1	ISL LIM homeobox 1	-3.74	1.09E-04	1.28E-05	cg26896762	92	4.78E-14	0.18	/	/
					cg21410991	818	1.24E-21	0.26	5.83E-03	0.16
MYH11	Myosin heavy chain 11	-2.92	1.63E-07	8.18E-09	cg17880199	28	2.57E-12	0.19	2.82E-02	0.17
					cg15488251	356	2.76E-02	0.05	/	/
PAX7	Paired box 7	-6.31	5.45E-15	5.90E-17	cg11428724	132	6.74E-22	0.44	7.52E-04	0.38
			6.21E-06	4.83E-07	cg07536847	268	8.93E-31	0.31	4.25E-04	0.22

a log_2_FC: log_2_ transformed fold change.

b FDR: Benjamini & Hochberg adjusted *p*–value (False discovery rate).

c TSS: transcription start site.

### Validation of The Hub Genes

**Figure 5:**
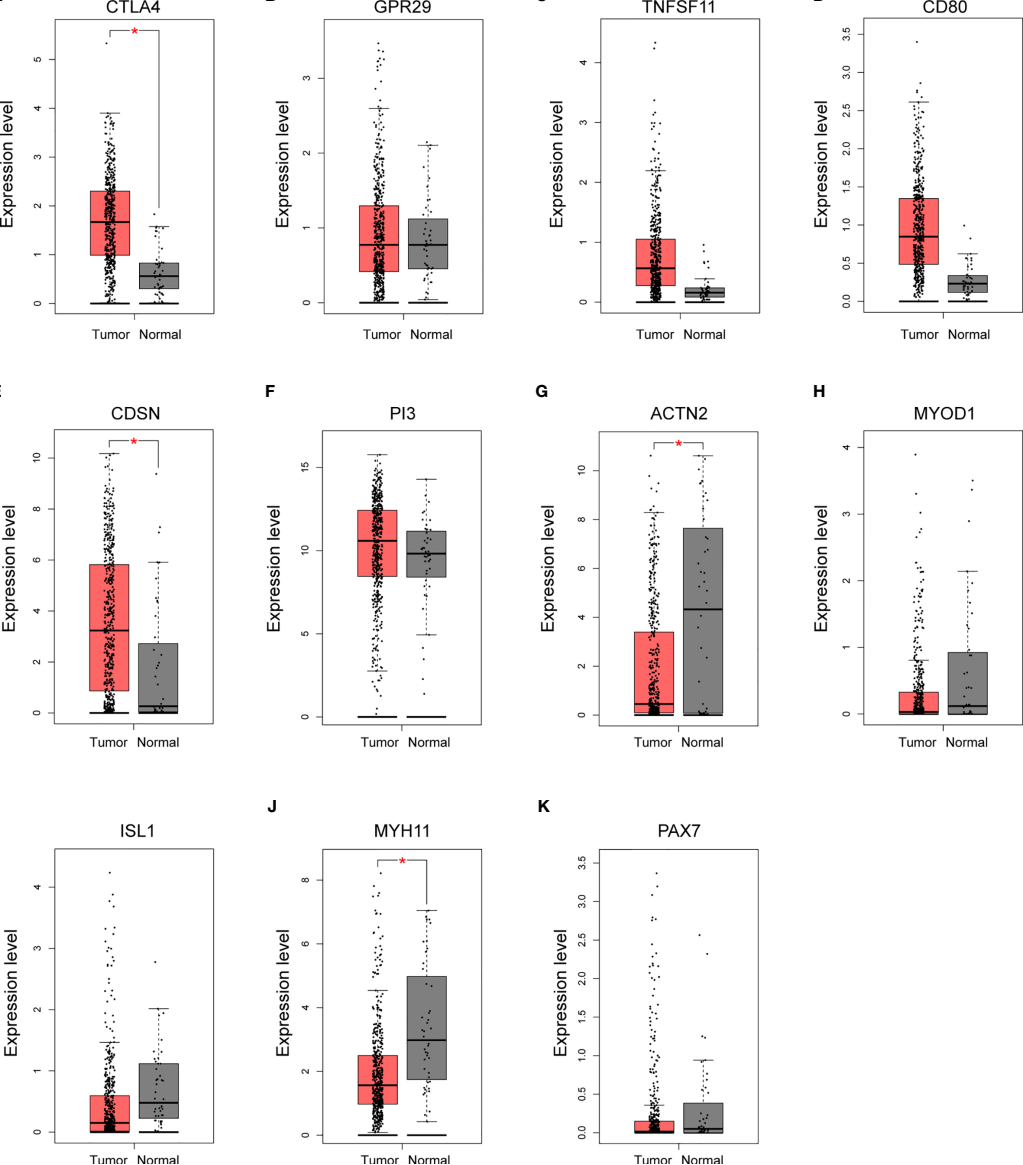
Validation of the expression of potential hub genes in the Cancer Genome Atlas (TGCA) database. **(A–K)** Box plots showing the relative expression levels of 11 potential hub genes across normal tissues (n = 44) and HNSC tumor samples (n = 519). Expression level indicates log_2_ (TPM + 1). TPM, Transcripts Per Million reads. *p < 0.05.

To evaluate the clinical relevance of these potential hub genes in OSCC, we assessed their expression levels in normal and tumor tissues using the GEPIA online tool. Since OSCC accounts for ~95% of all HNSC cases and a specific OSCC category is not included in TCGA database, similar to other studies, TCGA−HNSC data were obtained and used in this analysis (26). As shown in **Figures 5A–K**, the expression levels of four hub genes (CTLA4, CDSN, ACTN2, and MYH11) were significantly dysregulated in HNSC. The two hypomethylated hub genes, CTLA4 and CDSN, were significantly upregulated, and two hypermethylated hub genes, ACTN2 and MYH11, were downregulated in the HNSC tumor samples. All remaining hub genes, although without statistic strength, showed a correct trend with regard to gene expression in tumor tissues compared to those in NATs.

**Figure 6:**
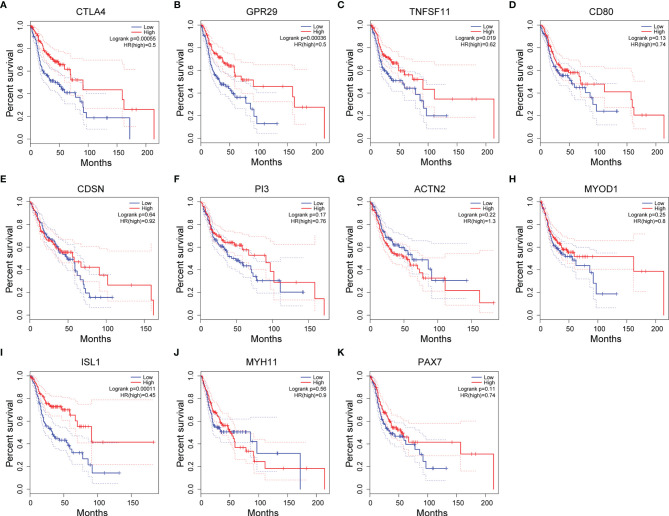
Associations between the expression level of hub genes and overall survival (OS) of HNSC patients. **(A–K)** Kaplan-Meier plots showing the OS of HNSC patients of 11 potential hub genes. Blue line: OS curve for patients with hub gene expression lower than the 25^th^ percentile. Red line: OS curve for patients with hub gene expression higher than the 75^th^ percentile.

In addition, we explored the prognostic value of these 11 hub genes by examining the association between the expression level of each gene and the OS of the HNSC cases using the Kaplan−Meier plotter (**Figures 6A–K**). The expression levels of four hub genes showed significant correlation with the HNSC clinical outcome. High expression levels of all three hypomethylated and upregulated hub genes (CTLA4, GPR29 and TNFSF11) presented a trend of association with longer OS (**Figures 6A–C**), while low expression of hypermethylated and downregulated hub gene ISL1 was found to be significantly associated with shorter OS (**Figure 6I**).

Considering the fact that HNSC datasets were used in these analyses, it may provide more insights if OSCC datasets could be specifically classified in TCGA and GEPIA to allow reexamination of the prognostic value of these genes.

## Discussion

The purpose of this study is to gain informative insights of the functional and clinically relevant profile of DNA methylation in OSCC. To achieve this goal, we took an integrated multi−omics approach to analyze the impact of DNA methylation on gene expression and biological pathways, then validated the association between the expression level of MeDEGs and the OS of HNSC patients in TCGA. Using this integrated approach, 226 DEGs with inverse corresponding DNA methylation changes and seven hub MeDEGs with potential clinical applications were identified. These findings may contribute to the understanding of the molecular mechanism as well as the development of potential DNA methylation biomarkers for OSCC.

There is strong evidence suggesting the involvement of aberrant DNA methylation in OSCC carcinogenesis. Compared to other epigenetic modification, DNA methylation attracts more attention since it is one of the earliest detectable neoplastic changes and is relatively reversible by chemical treatment. Microarray based methylomic evaluation provided a powerful tool for studying global changes of DNA methylation. To the best of our knowledge, eight groups have reported their studies identifying DNA methylation signatures in OSCC using a case−control study design, generating a sufficient amount of publicly available methylomic datasets (5, 9–15). Due to the inherent limitation of the high dimensionality of these huge data, an important question is how to select informative genes which are more biologically relevant to clinical outcomes and transfer these massive data into meaningful patterns with key genes and pathways. Recently, studies from two different groups have integrated transcriptomic and methylomic approaches to examine aberrant MeDEGs and their interrelationships in OSCC (16, 17). Since different inputs of publicly available datasets were used in these two studies, their findings barely overlap with each other. Therefore, more studies are needed to evaluate the power of this multi−omics approach and gain sufficient confidence for the key findings.

In our study, we generated RNA profiling from a stringently controlled collection of 17 paired samples of OSCC and NAT. These samples were similarly collected during the surgery procedure and immediately processed for RNA extraction to maximally preserve RNA integrity. A total of 778 upregulated genes and 2211 downregulated genes were identified through transcriptome analysis. Meanwhile, two methylation profiling GSE38532 and GSE46804, generated from same microarray platform and with similar paired case−control design, were obtained from public databases. Integrated multi−omics analysis revealed hypermethylation of 7.69% of under−expressed genes (170 of 2211) and hypomethylation of 7.20% of over−expressed genes (56 of 778) from these datasets. The finding that less than 10% DEGs are inversely associated with methylation modification is consistent with overall cancer associated MeDEGs from other studies (16, 27). The Illumina HumanMethylation 27 BeadChip covers 27,578 CpGs distributed genome−widely but specifically in proximal promoter regions. This supports the current knowledge of how DNA methylation regulates gene expression.

Our functional clustering analysis demonstrated that among all the DEGs, aberrant hypomethylation mainly occurs in genes involved in biological functions such as formation of the cornified envelope, extracellular matrix organization, anchoring fibril formation, regulation of cell adhesion, and intermediate filament cytoskeleton organization, all have been previously suggested to play roles in the development and progression of squamous cell carcinoma. Furthermore, hypomethylated MeDEGs are also enriched in the regulation of immune responses, including cellular response to bacterium, antimicrobial humoral response, and cytokine−mediated signaling pathway. This suggests the contribution of infective agents (such as previously reported HPV16 and Candida albicans) and immune dysfunction to OSCC, potentially through chronic inflammation related pathologies. For aberrantly hypermethylated DEGs, functions related to cell junction organization, regulation of membrane potential, and monocarboxylic acid metabolic process are observed to be enriched. Interestingly, multiple muscle related pathways like muscle system process, muscle structure development, muscle cell development, and muscle contraction are also found enriched. Therefore, consistent with previous reports, our study indicates that aberrant muscle function related cytoskeleton remodeling may assist the formation and progression of OSCC (28, 29). Other enriched functions like trans-synaptic signaling, regulation of ion transport, and phase I−functionalization of compounds is in agreement with our understanding that etiological factors like tobacco use, betel chewing, and alcohol consumption contribute to OSCC.

To understand the functional association among these MeDEGs, the PPI network of 56 upregulated MeDEGs and 170 downregulated MeDEGs were constructed using the STRING v11.0 database. Six highly connected hypomethylated genes (CTLA4, GPR29, TNFSF11, CD80, CDSN, and PI3) and five highly connected hypermethylated genes (ACTN2, MYOD1, ISL1, MYH11, and PAX7) were identified as hub genes from these networks. Hub genes are generally expected to play important roles in biological processes (30). Therefore, we further evaluated these hub genes for their correlations between observed dysregulation of DNA methylation and altered mRNA expression level in the TCGA database. Two hypomethylated hub gene (CTLA4 and CDSN) was found to be significantly upregulated, while two hypermethylated hub genes (ACTN2 and MYH11) showed obviously decreased expression level in the HNSC group. Therefore, our study identified four hub genes whose aberrant expressions were likely regulated by the mechanism of DNA methylation. Additionally, four hub genes showed important value in distinguishing the prognosis of HNSC patients. High expression levels of three individual hypomethylated hub genes (CTLA4, GPR29, and TNFSF11) presented a significant association with longer OS, while low expression of hypermethylated hub gene ISL1 was found to be clearly associated with shorter OS in HNSC patients. Therefore, these aberrantly methylated hub genes have potential diagnostic value and may become biomarkers and therapeutic targets for OSCC. Further studies with more specific OSCC datasets could provide more insights in the future.

CTLA4 (cytotoxic T-lymphocyte associated protein 4) is preferentially expressed in activated CD4^+^ T cells and constitutively expressed in CD4^+^Foxp3^+^ Treg cells (31, 32). It functions as a potent immune inhibitor by reducing the initiation of T-cell activation mediated by the interactions between antigen presenting cells (APCs) and T cells. Clinically, limiting CTLA4 function by antibody blockade was proved effective as a therapeutical approach to treat various cancers by the mechanism of boosting immune response against malignant tissues (33–36). In our study, we found that CTLA4 was not only significantly upregulated in patients with HNSC, but its high expression level was also associated with patients have better long-term survival. The median survival time was about 91 months for HNSC patients with high expression levels of CTLA4, compared with a median survival time of about 43 months for those patients with low expression levels of CTLA4. The beneficial effect of high expression of CTLA4 in HNSC, at first sight, seems contradictory to the well-known immune suppressive function, in other words, tumor−promoting role of CTLA4. However, the elevated expression of CTLA4 and beneficial effect of upregulated CTLA4 have also been previously noticed in other tumors (37, 38). To address the question of whether the upregulated CTLA4 in tumor tissues was originated from the increased immune infiltration, especially the infiltration of CTLA4 abundant CD4^+^ T cells, we estimated the abundances of six immune cell types (B cells, CD4^+^ T cells, CD8^+^ T cells, macrophages, neutrophils, and dendritic cells) in tumor and paired adjacent tissues by re−analyzing gene expression data using TIMER web tool (**Supplementary Figure S1**). Although the signals of certain immune cells were found higher in our OSCC samples (neutrophil and myeloid dendric cells) or TCGA HNSC samples (CD8^+^ T cell, neutrophil, and myeloid dendritic cells) compared to their corresponding NATs, the counts of CD4^+^ T cell infiltration were similar between tumor and paired adjacent tissues. Recently, a number of studies suggested that tumor cell-intrinsic CTLA4 might execute different functions, from inducing apoptosis to regulating cell proliferation, than that of T cells (39, 40). Here our results provide additional evidence that, depending on the context, the origin and the involvement of CTLA4 in tumorigenesis is complex.

We also identified two hypomethylated hub genes whose expression levels in tumor tissues showed positive association with the prognosis of HNSC patients, although their overall mRNA levels were not significantly changed in TCGA-HNSC samples compared to those in NATs. GPR29 (G−protein coupled receptor 29) is also known as CCR6 (CC chemokine receptor 6), a chemokine receptor that preferentially expressed by T cells, immature dendritic cells, and B cells. Its exclusive binding partner CCL20 (as known as macrophage inflammatory protein−3α, MIP-3α) is known to be steadily expressed by Th17 cells and secreted by intestinal epithelial cells as a response to local enteropathogenic infection (41). Therefore, CCL20−CCR6 axis plays crucial role in mucosal immunity. Apart from the immune-regulatory function, the elevated expression of CCR6 has been previously shown in various cancers, with complicated anti-cancer or pro-cancer potentials. TNFSF11 (tumor necrosis factor ligand superfamily member 11) is also known as receptor activator of nuclear factor kappa-B ligand (RANKL). It binds to its receptor RANK to regulate the differentiation, activation, and survival of osteoclast cells. While TNFSF11 is primarily known for its function in osteoclast regeneration, it has also been implicated in pathways like innate immune response, cell proliferation, and apoptosis. Here we report the first evidence that aberrant DNA methylation and aberrant expression level of TNFSF11 and GPR29 may play a role in the development of OSCC.

ISL1 (ISL LIM homeobox 1) is a LIM−homeodomain transcription factor. Aberrantly expressed ISL1 has been reported in various cancers, including gastric cancer, non-Hodgkin lymphoma, neuroblastoma, breast cancer, OSCC, etc. (42–44). Functionally, ISL1 has been implicated in many important biological pathways, such as tumorigenesis, cell invasion, apoptosis, and cancer immunity (42, 45). Although many studies indicated a tumor−promoting effect of elevated ISL1, Rajneesh et al. reported that hypermethylated and downregulated ISL1 was correlated with poorer survival in patients with breast cancer (46). In OSCC, Han et al. reported that ISL1 is significantly decreased and could be a potential biomarker of the disease (47). Consistently, in the present study, we identify the hypermethylated ISL1 as a key change in OSCC, indicating the prognostic value of ISL1 in OSCC.

Besides CTLA4, our study adds three additional hub genes with a significant and inverse correlation between the methylation pattern in promoter region and the mRNA expression level. The first one is ACTN2, for which the decreased expression level and elevated methylation status has already been reported in OSCC by another group (48). The second is MYH11, which encodes a myosin heavy chain family protein that is selectively expressed in smooth muscle. Similar to our observation, other studies have reported the downregulation of MYH11 in OSCC (49, 50). In colorectal cancer, survival analysis showed that low expression of MYH11 was significantly associated with poor prognosis (51, 52). Although the exact mechanism remains unclear, evidence suggests that the hypermethylation regulated interaction between DNMT3B and MYH11 may contribute to the tumor progression (53). The last is CDSN, which encodes corneodesmosin that is required for corneodesmosomes to maintain properly cornified squamous epithelia. Mutations in CDSN have been described in hereditary skin conditions such as hypotrichosis simplex of the scalp (HSS) and progressive loss of scalp hair (PSD) (54–57). In this study, we are the first to indicate hypomethylated and upregulated CDSN as a key change in OSCC.

## Conclusion

This study analyzed MeDEGs in OSCC using integrated multi-omics analysis and examined their related pathways and functions. From the networks of upregulated and downregulated MeDEGs, we identified the four hub genes (CTLA4, GPR29, TNFSF11, and ISL1) that are highly correlated with the OS of HNSC patients. In addition, we also verified four hub genes (CTLA4, CDSN, ACTN2, and MYH11) that their expression levels in tumor tissue might be regulated by the mechanism of DNA methylation. Therefore, this study provides a list of potential biomarkers and therapy targets for OSCC, as well as insights for unraveling the molecular mechanisms underlying this disease. However, it is worth noting the limitation of these discoveries generated from purely computational approach. Further prospective validation is warranted to characterize the clinical utilities and underlying mechanisms of these genes.

## Data Availability Statement

The datasets presented in this study can be found in online repositories. RNA sequencing data were available in NCBI GEO database (https://www.ncbi.nlm.nih.gov/geo; GSE186775). Microarray-based gene methylation profiling data set analyzed in this study are openly accessible in GEO database (https://www.ncbi.nlm.nih.gov/geo; GSE38532 and https://www.ncbi.nlm.nih.gov/geo; GSE46802).

## Ethics Statement

The studies involving human participants were reviewed and approved by the Ethics Committee of School of Life Sciences, Central South University. The patients/participants provided their written informed consent to participate in this study.

## Author Contributions

Conceptualization, ZW and DW. Methodology, ZW and DW. Software, ZW. Formal analysis, ZW. Investigation, ZW. Validation, ZW, XT, and DW. Resources, DW, HX, TS, and KX. Data curation, ZW. Writing—original draft preparation, ZW and DW. Writing—review and editing, ZW and DW. Visualization, ZW. Supervision, DW and KX. Project administration, ZW and DW. Funding acquisition, ZW and DW. All authors have read and agreed to the published version of the manuscript.

## Funding

This work is funded by The Science and Technology Innovation Program of Hunan Province, grant number 2019SK2124 and 2020RC2070. The funders had no role in the design of the study; in the collection, analyses, or interpretation of data; in the writing of the manuscript, or in the decision to publish the results.

## Conflict of Interest

The authors declare that the research was conducted in the absence of any commercial or financial relationships that could be construed as a potential conflict of interest.

## Publisher’s Note

All claims expressed in this article are solely those of the authors and do not necessarily represent those of their affiliated organizations, or those of the publisher, the editors and the reviewers. Any product that may be evaluated in this article, or claim that may be made by its manufacturer, is not guaranteed or endorsed by the publisher.

## Supplementary Material

Supplementary Figure 1 Immune infiltration estimation of tissue samples analyzed in this study. Immune infiltration of B cell **(A)**, CD4^+^ T cell **(B)**, CD8^+^ T cell **(C)**, Neutrophil **(D)**, Macrophage **(E)**, and Myeloid dendritic cell **(F)** were evaluated using TIMER2.0 web server. TCGA-HNSC samples and paired NATs were obtained from TCGA-HNSC project. NAT: Normal Adjacent Tissue. Statistical difference was evaluated using Student’s t test with Welch correction. **p* < 0.05, ***p* < 0.01, ****p* < 0.001, *****p* < 0.0001.
