# Supplementary figures and images for: Integrative Multi−Omics Analysis Reveals Candidate Biomarkers for Oral Squamous Cell Carcinoma
Source: Front Oncol. 2022 Jan 14;11:794146. doi: 10.3389/fonc.2021.794146 (PMC8795899; doi:10.3389/fonc.2021.794146)

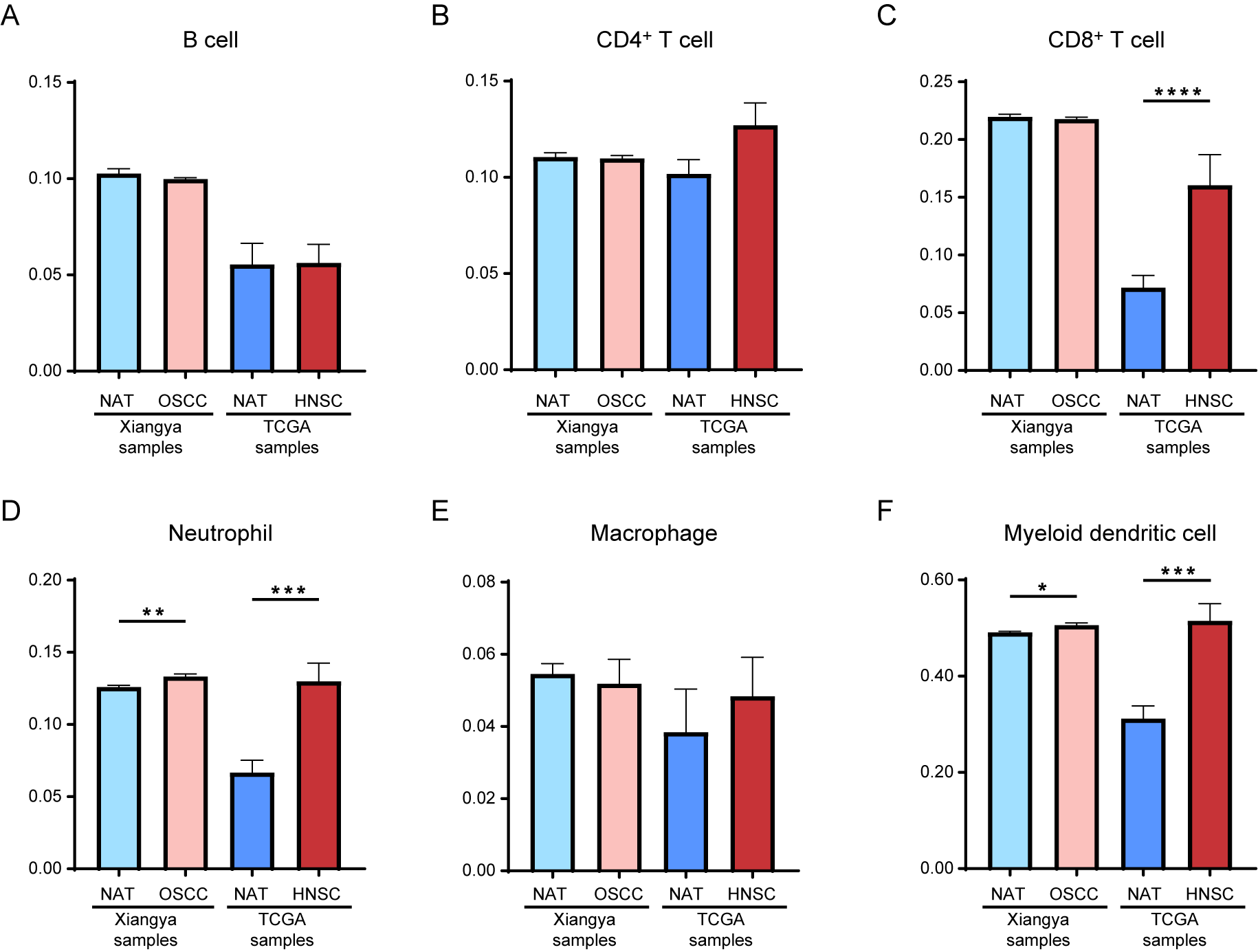

Supplement: Supplementary Figure 1 — Immune infiltration estimation of tissue samples analyzed in this study. Immune infiltration of B cell (A), CD4+ T cell (B), CD8+ T cell (C), Neutrophil (D), Macrophage (E), and Myeloid dendritic cell (F) were evaluated using TIMER2.0 web server. TCGA-HNSC samples and paired NATs were obtained from TCGA-HNSC project. NAT: Normal Adjacent Tissue. Statistical difference was evaluated using Student’s t test with Welch correction. *p < 0.05, **p < 0.01, ***p < 0.001, ****p < 0.0001. [file Image_1.tif]
